# Active Moderate-to-Severe Graves' Orbitopathy in a Patient With Type 2 Diabetes Mellitus and Vascular Complications

**DOI:** 10.3389/fendo.2018.00810

**Published:** 2019-01-17

**Authors:** Francesca Urselli, Gilda Pontieri, Livia Peschi, Alessia Liccardi, Anna Rita Ruggiero, Bernadette Biondi

**Affiliations:** Department of Clinical Medicine and Surgery, University of Naples Federico II, Naples, Italy

**Keywords:** hyperthyroidism, diabetes mellitus, Graves' orbitopathy, glucocorticoids, methotrexate, tocilizumab, radiotherapy

## Abstract

**Background:** Graves' orbitopathy (GO) is the main extrathyroidal manifestation of Graves' disease (GD). Diabetes mellitus (DM) has been reported to be a risk factor in patients with GO. Moreover, GO can be more frequent and severe in type 2 diabetes patients. High doses of intravenous glucocorticoids represent the first line treatment of moderate-to-severe and active GO according to the international guidelines. However, this therapy is contraindicated in uncontrolled diabetes and in patients with increased cardiovascular risk. Some anti-diabetic drugs can exacerbate GO. We reported the clinical case of an active and moderate-to-severe GO in a patient with uncontrolled type 2 DM and vascular complications.

**Case Report:** A 61-years-old patient came to our ambulatory for a recurrence of GD and a moderate-to-severe bilateral GO. The patient had uncontrolled type 2 DM during insulin therapy and a history of micro and macrovascular complications. At the physical examination, the clinical activity score was 5 and the severity of GO was moderate-to-severe. A blood sample showed overt hyperthyroidism and the persistence of anti-TSH receptor antibodies (TRAb) during treatment with methimazole. A computed tomography scan showed a moderate-to-severe bilateral exophthalmos. We discuss the benefit/risk of treatment of GO in our patient.

**Conclusion:** The available guidelines do not focus on the treatment of diabetic patients with uncontrolled diabetes and severe vascular complications, therefore our patient represents a difficult therapeutic challenge. The screening of thyroid function and the evaluation of GO could be useful in diabetic patients with autoimmune thyroid disease to perform a correct treatment of these disorders.

## Introduction

Graves' orbitopathy (GO) is an autoimmune disorder and represents the main extrathyroidal manifestation of Graves' disease (GD). It can also occur in patients with euthyroid or hypothyroid autoimmune thyroid disorders. The anatomical and histological findings of GO consist in soft tissues enlargement of the orbits due to an inflammatory infiltration (including T-cells, B-cells, and mast cells) of retro-orbital adipose tissue and extra-ocular muscles and proliferation of the connective tissue (1, 2).

The most common clinical features of GO are periorbital edema, conjunctival chemosis, exophthalmos, diplopia, corneal ulcerations, and upper-eyelid retraction; optic nerve compression can occur in severe cases (1–3).

There are standardized criteria for assessing the activity (active or inactive) and severity (mild, moderate-to-severe, and very severe) of GO to address its management (1, 3).

Smoking and thyroid dysfunction are well-recognized risk factors for GO, according to the guidelines of the European Group on Graves' Orbitopathy (EUGOGO) and the American Thyroid Association (1, 3). Therefore, in all patients with GD (also without GO) it is necessary to recommend not smoking. The restoration of euthyroidism is essential to avoid the exacerbation of GO (1, 3).

Diabetes mellitus (DM) has also been reported to be a risk factor in patients with GO (4). The association of autoimmune thyroid disease and type 1 diabetes mellitus is denominated as autoimmune polyglandular syndrome type 3 variant or APS3 (5). Moreover, GO can be more frequent and severe in type 2 diabetes mellitus (T2DM) patients (6).

We reported the clinical case of an active and moderate-to-severe GO in a patient with uncontrolled T2DM and vascular complications. As the first line treatment for moderate-to-severe and active GO, the EUGOGO guidelines recommend high doses of intravenous glucocorticoids (methylprednisolone); however, this therapy is contraindicated in uncontrolled diabetes and in patients with associated important cardiovascular risk factors (1).

The guidelines do not focus on the treatment of diabetic patients with severe vascular complications, therefore, the choice of a possible therapy for this category of patients is a difficult task.

## Case Presentation

**Figure 1:**
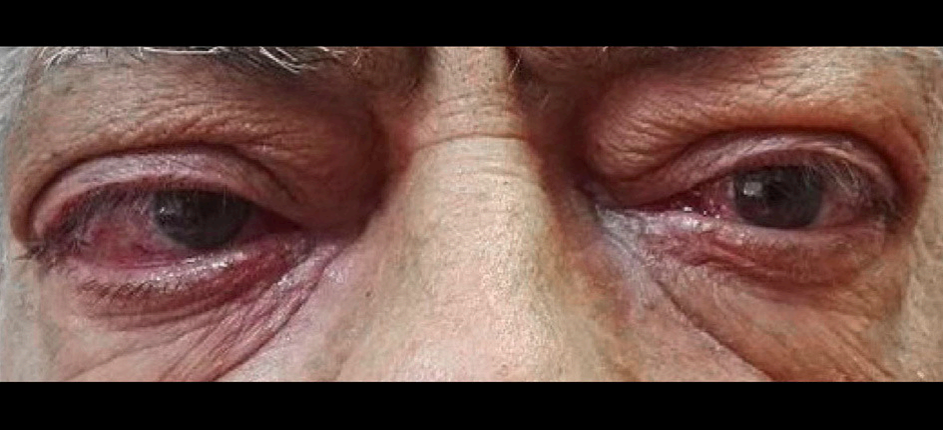
Clinical features of Graves' orbitopathy in our patient.

A 61-years-old patient came to our ambulatory for a recurrence of GD and a moderate-to-severe bilateral GO (Figure 1). He referred that GO developed at the first manifestation of GD in 2014 and worsened over time. The patient had type 2 DM with a metabolic glycemic decompensation (HbA1c 8.5%) during insulin therapy. He had been treated with thiazolidinediones (TZDs) many years before.

The patient had a history of micro and macrovascular diabetes complications, diabetic nephropathy with microalbuminuria and mild non-proliferative diabetic retinopathy. He mentioned that he had undergone an aorto-coronary bypass for ischemic heart disease and a foot amputation of the first metatarsal for diabetes.

At the physical examination, the thyroid was enlarged and the assessment of the GO activity showed the swelling of eyelids and caruncle and chemosis, confirming an active GO (CAS≥3) according to the European Thyroid Association guidelines. Severity of GO was moderate-to-severe for the presence of exophthalmos ≥3 mm above normal, diplopia, and severe soft tissue involvement. A blood sample showed overt hyperthyroidism and the persistence of thyrotropin receptor antibodies (TRAb) during treatment with anti-thyroid drugs (Table 1). Thyroid ultrasound showed an increased thyroid volume and vascularization. A computed tomography scan showed a bilateral exophthalmos with a thickening of the ocular extrinsic musculature.

**Table 1:** Thyroid function at our first evaluation.

		**Reference range**
TSH	0.01 μU/ML	(0.41–4.30)
FT3	7.6 PG/ML	(3.0–4.70)
FT4	23.1 pmol/l	(9–20)
TRAB	26.8 U/L	(<1.75)
ABTG	780 U/ML	(0–60)

We analyzed the literature data to choose the best treatments of GO for our diabetic patient.

## Discussion

Type 1 diabetes mellitus (T1DM) is a risk factor for GD for the associated common genetic susceptibility. Moreover, the incidence of optic neuropathy (a very severe GO) is higher in patients with GO and type 1 DM (33.3%), compared to patients with GO without DM (3.9%). These data could be explained by the low oxygenation of the optic nerve and his compression by the expansion of extraocular muscles due to microvascular complications of DM (4, 7). A strong link between GD and type 1 DM, but not with type 2 DM was reported in an Italian study (6). However, GO was more severe in patients with long term T2DM and associated microvascular and macrovascular complications. Insulin-like growth factor I (IGF1) bioavailability was found to be increased in patients with T2DM and GO because of the insulin-resistance; the compensatory hyperinsulinemia probably reduced the IGF-1 binding proteins 1 and 2 (6). Furthermore, an overexpression of IGF-1 receptor was found in orbital pre-adipocytes/fibroblast. All these factors could contribute to the increase adipogenesis, inflammation, and overproduction of hyaluronan in orbital tissue (6).

Treatment of T2DM can affect the onset and progression of GO. In fact, TZDs, which are potent agonists of peroxisome proliferator activated receptor-γ (PPAR-γ), can exacerbate GO. PPAR-γ is located in the adipose tissue and can increase retrobulbar fat and stimulate the TSH receptor expression in orbital fibroblasts (6, 8). Therefore, TZDs can contribute to the pathogenesis of GO in T2 diabetic patients.

On the contrary, a recent *in vitro* study (carried out on fibroblast cultures taken from GO patients undergoing orbital decompression) demonstrated a possible therapeutic effects of biguanides by inhibiting hyaluronan synthesis and pro-inflammatory molecule production in orbital fibroblasts (9). Moreover, metformin and phenformin significantly inhibit the adipogenic pathway during the differentiation of orbital fibroblasts (9).

The current guidelines do not recommend specific therapeutic options for GO in patients with uncontrolled DM and vascular complications, even in those with cardiovascular comorbidity (1). Intravenous high-dose of glucocorticoids, alone or in association with ciclosporin, are considered the treatment of choice for moderate-to severe active GO for their efficacy (1). However, these drugs are contraindicated in uncompensated diabetes and in patients with severe hypertension and coronary heart disease. Orbital radiotherapy, which is recommended as a second-line treatment for GO can be used alone or in combination with glucocorticoids (1). This treatment improves diplopia and ocular motility and is considered relatively safe. However, diabetic retinopathy and uncontrolled hypertension represent an absolute contraindication for this treatment because of the increased worsening of retinopathy (1, 10, 11).

The off-label use of rituximab (RTX) is also considered a second-line treatment by the EUGOGO guidelines (1). RTX is a monoclonal antibody anti-CD20 commonly used in the treatment of autoimmune disease and B cell lymphomas. Two randomized clinical trials evaluated the efficacy of RTX (12, 13) and showed conflicting results. RTX was effective in patients with active moderate-to-severe GO compared with intravenous glucocorticoids in the study by Salvi et al. in which 32 patients with thyroid-associated orbitopathy were treated with intraorbital low doses of RTX vs. high doses of systemic glucocorticoids (12). These results highlight the efficacy of RTX in reducing orbitopathy CAS and severity. However, important side effects (such as myocardial infarction and arrhythmias) have been reported in cardiac patients (14). Moreover, some cases of optic neuropathy have been described in patients with GO treated with RTX (15).

There are some data on the potential use of methotrexate (MTX), an immune suppressive drug that inhibits folic acid synthesis and is used for the treatment of several autoimmune diseases. MTX may have a potential role in GO for its immunosuppressive properties (16). One study showed its efficacy in the treatment of GO as a corticosteroid-sparing agent in patients previously treated with prednisone (17).

A successful treatment with tocilizumab has been reported in two patients with GO (18). Tocilizumab is a IgG monoclonal antibody targeting the IL-6 receptor; it is used for the treatment of rheumatoid arthritis not-responsive to conventional anti-rheumatic drugs. As demonstrated by *in vitro* studies, pro-inflammatory cytokine IL-6 and TSH receptors mutually stimulate their expression in orbital fibroblasts of patients with GO (19, 20). Tocilizumab interrupts the inflammatory process by blocking the IL-6 receptors (20); this IL-6 signal inhibition can have positive effects in diabetic subjects. TSH and IGF-1 work interdependently in the GO pathogenesis, and antibodies recognizing and activating the IGF-IR signaling have been detected in patients with GD (21). Moreover, an inverse correlation was found between IGF-IR and the CAS (22). Teprotumumab, a IGF-1 receptor inhibitory antibody, has been evaluated in a recent multicenter double-masked randomized placebo-controlled trial in patients with GO. This treatment improved proptosis and reduced the CAS (22). However, hyperglycemia was observed in some patients with diabetes (22).

All of these options were not considered suitable for our patient because of the potential side-effects. Moreover, thyroidectomy was contraindicated for the severe cardiac conditions.

Our patient had a mild non-proliferative diabetic retinopathy. Therefore, we discussed with him about the benefit/risk of treatment of GO and decided to perform low-doses of intravenous glucocorticoids (1,500 mg in 6-weeks) administration (after monitoring glycemic and cardiac conditions) only in the event of further exacerbation of his ocular symptoms, eventually associated with fractionated low-dose orbital radiotherapy (1 Gy per week over a 20-weeks period) (10, 23, 24). This scheme of 1 Gy per week over a 20-weeks period was effective and better tolerated compared to 2 Gy daily over 2-weeks in patients with moderately severe GO, showing lower rates of side effects compared to high RX dose (23).

## Conclusions

Diabetes mellitus can be considered a risk factor for GO in patient with Graves' disease or autoimmune thyroid disorders. Considering the high prevalence and severity of GO in patients with T1 and T2DM, the screening of thyroid function and the evaluation of orbitopathy is useful in diabetic patients to perform a correct treatment of these disorders. Caution should be placed for the choice of anti-diabetic drugs in patients with GO and DM.

Based on the above mentioned considerations, our patient represents a difficult therapeutic challenge. In the future, international guidelines could help clarify the therapeutic options for GO in patients with uncontrolled DM and associated vascular complications.

## Author Contributions

All authors listed have made a substantial, direct and intellectual contribution to the work, and approved it for publication.

### Conflict of Interest Statement

The authors declare that the research was conducted in the absence of any commercial or financial relationships that could be construed as a potential conflict of interest.
